# Simultaneous radical nephroureterectomy and transurethral distal ureter balloon occlusion and detachment

**DOI:** 10.1186/1477-7819-12-345

**Published:** 2014-11-15

**Authors:** Luigi Cormio, Oscar Selvaggio, Giuseppe Di Fino, Paolo Massenio, Francesca Sanguedolce, Giuliano Ciavotta, Vito Mancini, Giuseppe Carrieri

**Affiliations:** Department of Urology and Renal Transplantation, University of Foggia, Viale L Pinto 1, 71121 Foggia, Italy; Department of Pathology, University of Foggia, Foggia, Italy

**Keywords:** Urothelial carcinoma, Upper urinary tract, Nephroureterectomy

## Abstract

**Background:**

Distal ureter bladder cuff (DUBC) excision is an essential part of radical nephroureterectomy (RNU) but there is no agreement on the ideal surgical technique to achieve it. We describe a novel technique for endoscopic DUBC excision during RNU that complies with the oncological principle of preventing spillage of tumor cells, by occluding the distal ureter before its excision, while shortening surgical time, and by avoiding repositioning the patient.

**Methods:**

Between June 2010 and May 2012, 10 patients underwent simultaneous open RNU and transurethral distal ureter balloon occlusion and detachment using a flexible cystoscope (f-TUDUBOD) in lumbotomy position. After having ruled out the presence of a concomitant bladder tumor, one surgeon used a flexible cystoscope to occlude the affected ureter with a 5Fr Fogarty catheter and circumferentially incised the orifice until detaching it from the bladder with a boogie electrode or a Holmium laser; meanwhile, two other surgeons performed open RNU through a lumbotomic approach. Data were compared with those of patients who had previously undergone open RNU after TUDUBOD.

**Results:**

Mean surgical time for simultaneous open RNU and f-TUDUBOD was 113.4 ± 29.2 minutes, significantly shorter (*P* <0.01) than that for open RNU after TUDUBOD (154.2 ± 26.4 minutes). There were no complications. Surgical margins were always negative; at mean follow-up of 31.1 months, there was no recurrence in the perivesical space and a 20% (2/10) bladder recurrence rate comparing favorably with that (23.1%) observed at 30-month follow-up in patients who had undergone open RNU after TUDUBOD.

**Conclusions:**

Simultaneous open RNU and f-TUDUBOD proved to be feasible and to represent a safe and effective means of shortening surgical time, with obvious clinical and economical benefits.

## Background

Radical nephroureterectomy (RNU) with distal ureter bladder cuff (DUBC) excision is the standard treatment for high-risk non-invasive and invasive upper tract transitional cell carcinoma (UT-TCC), but there is no agreement on the ideal technique for DUBC excision [1–3]. The extravesical approach avoids repositioning the patient but violates the golden rule of excising the DUBC under direct vision; conversely, the transvesical and the endoscopic approach allow DUBC excision under direct vision but require the patient to be repositioned, thus being time-consuming. Independent of the DUBC excision technique, the procedure should comply with the oncological principle of preventive ureteral occlusion and *en bloc* specimen removal to avoid spillage and consequent seeding of tumor cells.

To comply with the oncological principle of preventing spillage of tumor cells, we recently described a technique for transurethral distal ureter balloon occlusion before detachment called TUDUBOD [4]. Briefly, the patient is placed in the dorsal lithotomy position and the distal ureter occluded with a balloon catheter to prevent spillage of tumor cells outside the bladder while excising the DUBC with a resectoscope; after having completed this phase, the patient is turned to the lumbotomy position for open RNU.

To overcome the problem of prolonged surgical time due to the use of two different patient positions, we subsequently attempted to carry out the transurethral procedure with the patient in the lumbotomy position, simultaneously to open RNU, using a flexible cystoscope (f-TUDUBOD). The present study therefore describes the technique for simultaneous open RNU and f-TUDUBOD and evaluates its efficacy in shortening surgical time.

## Methods

Following Institutional Review Board approval, 10 patients underwent simultaneous open RNU and f-TUDUBOD between June 2010 and May 2012. All patients signed a written informed consent.

**Figure 1:**
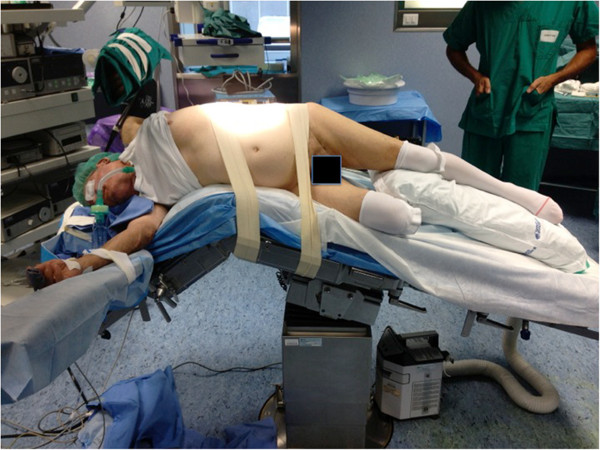
**The male patient is placed in the standard lumbotomy position.**

**Figure 2:**
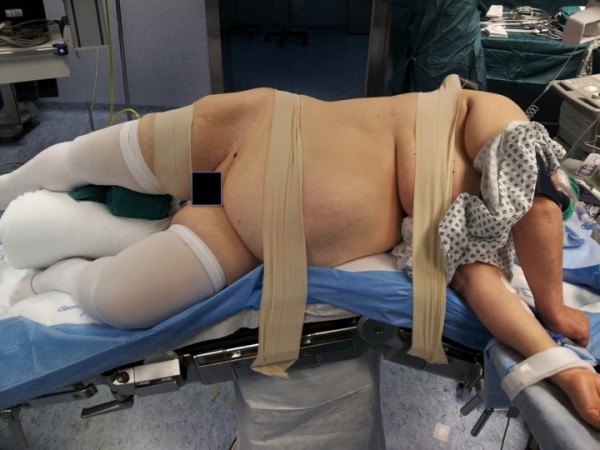
**In female patients, a large pillow is placed between the two legs at the level of the foot of the contralateral and the knee of the homolateral leg, to provide an easier access to the urethra.**

After general anesthesia, the patient was placed in the standard lumbotomy position (Figure 1); in females, a large pillow was placed between the patient’s legs at the level of the foot of the contralateral and the knee of the homolateral leg, to provide an easier access to the urethra (Figure 2). The surgeon performing open RNU and the scrub nurse stood posterior to the patient, whereas his assistant and the surgeon performing the transurethral procedure stood anterior to the patient; the endoscopic equipment was placed cranially posterior to the patient.

**Figure 3:**
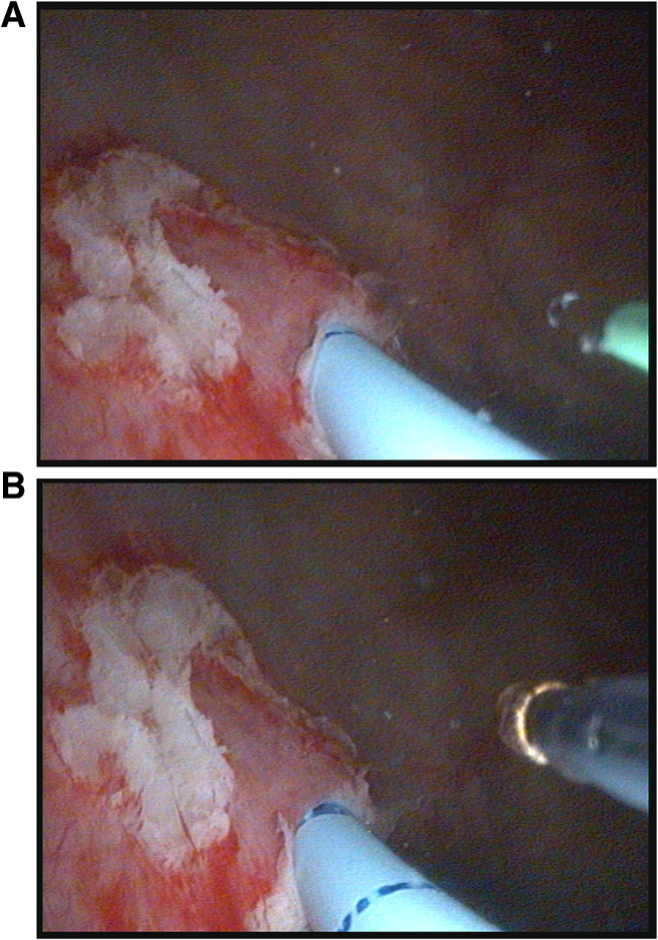
**Following insertion of a 5Fr Fogarty catheter into the affected ureter, the DUBC is circumferentially incised up to the perivesical fat to detach it from the bladder using a 365 μm Holmium laser fiber (A) or a 5Fr boogie electrode (B).**

Using a flexible cystoscope (f-TUDUBOD), a 5Fr Fogarty occlusion catheter was inserted into the affected ureter; the distal end of the Fogarty catheter was cut to remove it from the instrument and a connector used to inflate the balloon with 1 ml saline for complete ureteral occlusion [4]. The flexible cystoscope was re-introduced alongside the Fogarty catheter and the DUBC circumferentially incised up to the perivesical fat to detach it from the bladder using a 365 μm Holmium laser fiber or a 5Fr boogie electrode (Figure 3). An 8Fr Nelaton catheter placed alongside the flexible cystoscope provided continuous bladder drainage during the procedure. Finally, a 22Fr Foley urethral catheter was left indwelling. Meanwhile, two other surgeons performed open RNU through a lumbotomic approach as previously described [4].

Our prospective Institutional Review Board-approved database for upper tract transitional cell carcinoma (UT-TCC) was used to compare data of these patients with those of patients who had previously undergone open RNU after TUDUBOD. Continuous data were reported as mean ± standard deviations (SD) and analyzed by the one-way analysis of variance (ANOVA) or the Mann-Whitney test, depending on their normal or non-normal distribution, respectively. Differences in rates were assessed using Fisher’s exact test. Statistical analysis was carried out using commercially available software (MedCalc version 11.1.0.0, Mariakerke, Belgium). Significance was set at *P* <0.05.

## Results

Patients’ data are summarized in Table 1. All patients presented with hematuria and were diagnosed with UT-TCCs by computed tomography (CT) scanning; 9 were scheduled for RNU, as they had large (>3 cm) UT-TCCs not suitable for ureteroscopic treatment, whereas 1 with a 1-cm renal pelvis UT-TCC was scheduled for RNU following ureteroscopic removal that showed a high-grade pT1 cancer. Two patients had a history of a low-grade pTa bladder cancer, which had never recurred following transurethral resection performed 16 and 47 months before, respectively. Preoperatively, all patients had undergone flexible cystoscopy to rule out concomitant bladder cancer.

**Table 1:** **Patients’ data**

Number of patients	10
Mean age (range)	68.2 years (43 to 87)
Sex (M/F)	7/3
Side (R/L)	7/3
Tumor site	
pyelocalyceal	6
proximal ureter	2
both	2
Mean surgical times	
f-TUDUBOD	22.2 minutes (20 to 30)
all procedure	113.4 minutes (95 to 140)
Mean hospital stay (range)	6.5 days (5 to 10)
Complications (%)	0
Tumor grade	
Low-grade	3
High-grade	7
Tumor stage	
Ta	5
T1	2
T2	0
T3	3
Nx/N0/N+	6/4/0
Surgical margins	
negative	10
positive	0
Mean follow-up (range)	31.1 months (21 to 44)
Bladder recurrences	2 (20.0%)
DUBC excision site or extravesical recurrences	0

Mean surgical time for simultaneous open RNU and f-TUDUBOD was 113.4 ± 29.2 minutes with a mean surgical time for f-TUDUBOD of 22.2 ± 6.8 minutes. The laser fiber was used in five cases and the boogie electrode in the other five, with no difference in mean surgical time between the two energy sources. Mean postoperative hospital stay was 6.5 days (range 5 to 10). No patient suffered complications (Table 1).

Final pathologic tumor staging and grading is described in Table 1. Surgical margins were always negative for both kidney specimens and DUBCs. One patient with high-grade pT3 disease and lymphovascular invasion received adjuvant chemotherapy. At mean follow-up of 31.1 months, there was no recurrence in the perivesical space and a 20% (2/10) bladder recurrence rate, comparing favorably with that (23.1%) observed at 30-month follow-up in patients having undergone open RNU after TUDUBOD. However, bladder recurrences did occur in patients with no history of previous bladder cancer.

Comparing data with those of patients who had previously undergone open RNU after TUDUBOD [4], simultaneous open RNU and f-TUDUBOD was associated with a significantly shorter surgical time (113.4 ± 29.2 versus 154.2 ± 26.4 minutes; *P* <0.01). With both techniques, surgical margins were always negative and there was no recurrence in the perivesical space as assessed by yearly follow-up CT scanning. Finally, the 20% bladder recurrence rate at 31.1 months follow-up for simultaneous open RNU and f-TUDUBOD compared favorably with that (23.1%) observed at 30-month follow-up in patients having undergone open RNU after TUDUBOD.

## Discussion

RNU is a technique under continuous refinement. Regarding the removal of kidney and proximal ureter, the open and laparoscopic approaches seem to be equivalent in terms of efficacy but the potential functional advantages of the laparoscopic approach should be weighed against the possibility of the accompanying pneumoperitoneum increasing the risk of tumor spillage [1].

Much more controversial is the management of the DUBC [3]. The extravesical approach, though allowing preventive ureteral occlusion, violates the golden rule of excising the DUBC under direct vision; as a matter of fact, *the extravesical approach may result in incomplete excision if too close to the orifice, or damage to the contralateral ureter if too extended.* The open transvesical approach guarantees DUBC excision under direct vision after preventive ureteral occlusion but violates bladder integrity in both cystotomy and DUBC excision sites in a patient with a urinary tract TCC. Moreover, it is invasive, difficult in obese patients or those who have undergone previous pelvic surgery, and is associated with significant surgical time and postoperative patient discomfort.

The endoscopic approach provides an attractive, minimally invasive means of performing DUBC excision under direct vision, providing preventive ureteral occlusion. As a matter of fact, the risk of tumor cell spillage after the classical ‘pluck’ technique of transurethral resection of the intramural ureter without preventive ureteral occlusion is documented by several cases of extravesical recurrences [5]. Additionally, coagulation of the ureteral orifice before circumferential incision and detachment of the intramural ureter was found, in a recent large retrospective multicentric study, to be associated with a higher intravesical recurrence rate as compared to the extra- or transvesical approaches [3]; thus pointing out the need for complete ureteral occlusion before endoscopic DUBC excision.

In the last two decades, several techniques for complete ureteral occlusion before endoscopic DUBC excision have been described. Transurethral partial circumferential incision of the intramural ureter, ligation of the orifice with an endoloop through 2 needlescopic 2 mm ports placed transvesically, and transurethral completion of DUBC detachment has been shown to be oncologically safer than ‘pluck’ DUBC excision or laparoscopic extravesical stapling [6]. This, and other similar techniques [7, 8] of transvesical laparoscopic DUBC excision, however, are technically difficult and also violate bladder integrity, making transurethral techniques more urologist-friendly and oncologically sound means of ureteral occlusion and DUBC excision.

Transurethral partial circumferential incision of the intramural ureter, ligation of the mushroom-shaped ureteral stump with a PDS endoloop [9] or a Hem-o-lok clip placed through the straight working channel of a rigid nephroscope [10] before completing DUBC excision, is relatively fast and easy to perform. However, care must be taken not to incise the bladder wall through to the perivesical fat before the ureteral orifice is occluded. To avoid such a problem, injection of fibrin sealant through a ureteral catheter [11] or placement of a Fogarty catheter [4] have been suggested as simple and safe means of occluding the distal ureter before DUBC excision. However, such technical refinements, though increasing ease and oncological safety of RNU, do not overcome the problem of such a procedure being time-consuming, requiring two different steps and patient repositioning between these steps.

The use of a flexible cystoscope and a 5Fr electrode for transurethral DUBC excision (without occlusion) at the end of laparoscopic RNU has been described as a mean of avoiding patient repositioning and, secondarily, providing a ‘gentler’ approach to the ureter in patients with a large prostatic middle lobe [12].

The present study demonstrates the feasibility of simultaneous open RNU and f-TUDUBOD. There was no difference in using the boogie electrode or the Holmium laser fiber; the latter had the advantage of working in saline but the disadvantage of a more difficult coagulation. Whatever the energy source, f-TUDUBOD was obviously somewhat more technically demanding than standard TUDUBOD with a resectoscope. The comparison with open RNU after TUDUBOD showed that simultaneous open RNU and f-TUDUBOD provided a significant reduction of surgical time, by eliminating the mean 40 minutes needed for TUDUBOD and subsequent patient repositioning [4], and yielded similar oncologic outcomes.

Potential study limitations include the relatively small number of patients, the relatively short mean follow-up, and the technique being unsuitable for tumors located close to the ureteral orifice; however, this study aimed at determining the feasibility of simultaneous open RNU and f-TUDUBOD and its efficacy in shortening surgical time. Given the promising results obtained in this initial series, we feel that this technique deserves further investigation.

## Conclusions

Simultaneous open RNU and f-TUDUBOD proved to be feasible and to represent a safe and effective means of shortening surgical time, with obvious clinical and economical benefits. Further studies are needed to determine the feasibility of simultaneous f-TUDUBOD and laparoscopic RNU, as well as long-term oncologic results of this technique.
